# Congenital pancreaticobiliary anomalies in an urban medical center in the United States

**DOI:** 10.1002/jgh3.12418

**Published:** 2020-10-15

**Authors:** Choichi Sugawa, Ashley Culver, Naresh Sundaresan, Charles E Lucas

**Affiliations:** ^1^ Michael and Marian Ilitch Department of Surgery Wayne State University School of Medicine Detroit, Michigan USA

**Keywords:** choledochal cyst, endoscopic retrograde cholangiopancreatography, pancreas divisum, pancreaticobilary anomalies, pancreaticobiliary maljunction, treatment

## Abstract

**Background and Aim:**

Pancreaticobiliary anomalies are rare and often present with cryptic signs and symptoms, thus delaying appropriate treatment.

**Methods:**

Endoscopic retrograde cholangiopancreatography (ERCP) was used to define pancreaticobiliary anomalies. A retrospective review was performed of 5522 ERCPs conducted at a tertiary care center from 1972 to 2015.

**Results:**

There were 249 (4.5%) patients with pancreaticobiliary anomalies, including 179 patients with pancreas divisum (PD), 44 patients with choledochal cyst (CC) (Todani's classification Type I: extrahepatic cyst 31, Type III; choledochocele 9, Type V: Caroli's disease 4), 20 patients with anomalous pancreaticobiliary ductal union (APDU), and 6 patients with other abnormalities. Of 179 patients with pancreas divisum, 8 (4.5%) required minor sphincterotomies for multiple unexplained acute pancreatitis. Of the 31, 15 (48%) Type I CC patients underwent an operation. In patients with Type III CC (choledochocele), seven of the nine were treated by endoscopic sphincterotomy, and two patients were treated by surgery. Four patients with Type V CC (Caroli's disease) were managed nonoperatively. Of the 20 patients with APDU, 8 (40%) required operative intervention. Six patients were found to have other anomalies: two with pancreas bifidum, one with a duplication of the gallbladder, one with a cystic duct diverticulum, one with an annular pancreas, and one with an abnormal cystic duct origin. These patients were treated based on their etiology.

**Conclusion:**

Pancreaticobiliary anomalies are rare and can be defined using ERCP. The appreciation of these abnormalities is important for the proper diagnosis and treatment of these rare biliary and pancreatic disorders.

## Graphical abstract

Pancreaticobiliary anomalies were analyzed using endoscopic retrograde cholangiopancreatography (5522 patients) in an urban medical center in the United States. A total of 249 (4.5%) patients were found to have a pancreaticobiliary anomaly. There were 179 patients with pancreas divisum, 44 patients with choledochal cyst, 20 patients with anomalous pancreaticobiliary ductal union, and 6 patients with other abnormalities.

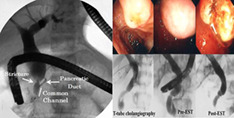

## Introduction

Pancreaticobiliary anomalies represent variants of pancreatic and biliary ductal development. Examples of these include pancreatic divisum (PD), choledochal cysts (CC), anomalous pancreaticobiliary ductal union (APBDU), and other congenital pancreaticobiliary anomalies. Patients affected by these anomalies often present with cryptic signs and symptoms that can be missed or are poorly understood, thus delaying or even preventing appropriate treatment. In addition, pancreaticobiliary anomalies may portend an increased risk of carcinogenesis in certain patient populations. A broader appreciation of these anomalies will facilitate a definitive and timely diagnosis, which can be obtained by endoscopic retrograde cholangiopancreatography (ERCP). This study is designed to elucidate the subtle nuances of these anomalies and to define optimal treatment. These results are based on the 46‐year (43‐year) experiences, from 1972 to 2015, of a single senior surgical endoscopist at a tertiary care center.

## Methods

A retrospective review of 5522 ERCPs performed at a tertiary care urban medical center by a single surgical endoscopist from 1972 to 2015 was performed. A total of 249 (4.5%) patients were found to have a pancreaticobiliary anomaly. These anomalies were documented and categorized as PD; CC, which were divided into five types according to Todani's modifications of the Alonso‐Lej classification;^1^, ^2^ APBDU; and other congenital pancreaticobiliary anomalies (Table 1). A retrospective chart review was carried out to obtain in‐depth information regarding patient information and therapeutic interventions with the approval of the Institutional Review Board at Wayne State University under the IRB# 045616MP4E.

**Table 1:** Congenital pancreaticobiliary anomalies (249/5572 = 4.5%)

Disease		Cases
Pancreas divisum		179 (3.3%)†
Choledochal cyst		
Cystic dilation of CBD (Type I)	31	
Choledochocele (Type III)	9	
Caroli's disease (Type V)	4	44 (0.8%)†
Anomalous pancreaticobiliary duct union		20 (0.36%)†
Other anomalies		
Pancreas bifidum	2	
Double gallbladders	1	
Diverticulum of the cystic duct	1	
Annular pancreas	1	
Abnormal cystic duct origin	1	
Total		249 (4.5%)

† Duplicate.

## Results

There were 249 patients with pancreaticobiliary anomalies (Table 1), including 179 patients with PD, 44 patients with CC, 20 patients with APBDU, and 6 patients with other pancreaticobiliary anomalies (2 patients with pancreas bifidum and 1 patient each with duplication of the gallbladder, cystic duct diverticulum, annular pancreas, and abnormal cystic duct origin).

### *Pancreas divisum*

The 179 patients (3% having ERCP) with PD ranged in age from 5 to 89 years (Table 2). Forty‐five percentage of patients were male. There were 148 patients with complete PD and 31 patients with incomplete divisum. There were 59 patients (33%) who had clinical evidence of pancreatitis. Twenty‐six patients (14.5%) presented with cholelithiasis or choledocholithiasis (Table 2). Five patients were found to have CC, four patients (2.2%) were found to have common bile duct cancer, three patients (1.7%) had ampullary cancer, and two patients (1.1%) had pancreatic cancer. Two of the patients (1.1%) with PD had coexisting APBDU. Of the 179 patients with PD, 8 (4.5%) underwent minor sphincterotomy (4 endoscopic, 3 surgical, and 1 both endoscopic and surgical). The other operations were for associated conditions such as biliary stones or malignancy.

**Table 2:** Characteristics of patients with pancreas divisum

179 cases	(179/5572 = 3.2%)
Age: 5–89 years	(mean: 47.7 years)
Male 81	Female 98
Complete PD: 148 (83%)	Incomplete PD: 31
Associated findings	
Pancreatitis	59 (33%)
Cholecyst/choledocholithiasis	26 (15%)
Choledochal cyst	5†
Common bile duct cancer	4
Ampullary cancer	3
Pancreatic cancer	2
APDU	2†

† Duplicate.

### *Choledochal cysts*

CCs are divided into five types according to Todani's modifications of the Alonso‐Lej classification (Table 1).^1^, ^2^ Type I refers to extrahepatic bile duct dilation and is also subcategorized into columnar (Ia), cystic (Ib), and spindle types (Ic).^1^, ^3^, ^4^ Type II is a diverticulum in the extrahepatic duct; Type III is a choledochocele; and Type IV has two types: Iva, which is multiple cysts in the intra‐ and extrahepatic ducts, and IVb, which is multiple cysts in the extrahepatic duct only. Finally, Type V, also known as Caroli's disease, demonstrates multiple intrahepatic duct cysts.^1^, ^2^

**Figure 1:**
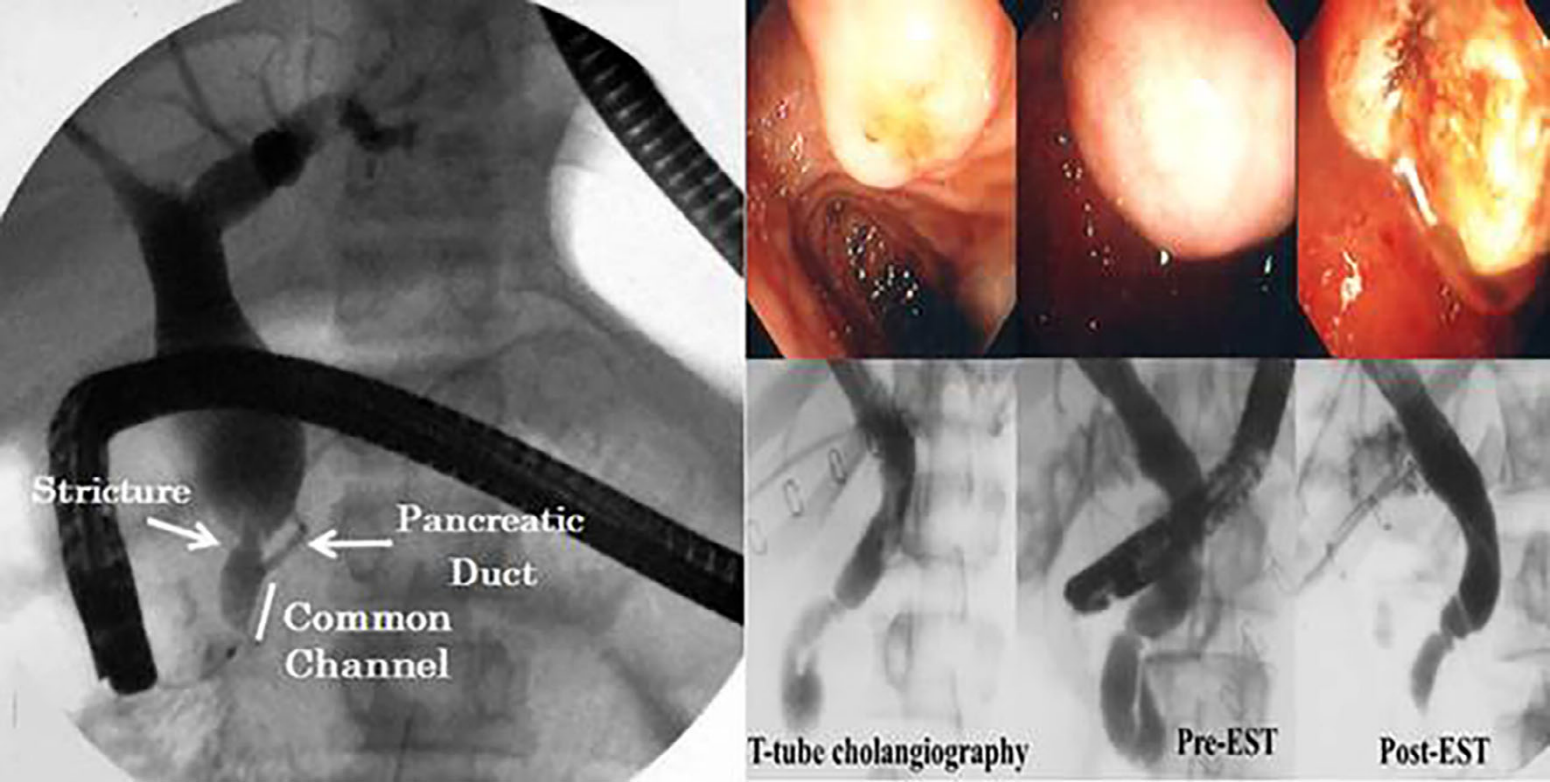
(a) Choledochal cyst (type Ic). Stricture of bile duct (arrow) just above the common channel. (b) Choledochocele (type III): T‐tube could not be removed after cholecystectomy and common bile duct exploration (left). ERCP showed a choledochal cyst before endoscopic sphincterotomy (EST) (center). The bile drained well after EST (right).

Todani's Type I extrahepatic bile duct cyst (Fig. 1a) was found in 31 (0.56%) of the 5522 patients having ERCP (Table 3). Their ages ranged from 8 to 80 years, and there were 10 men and 21 women. Duct‐graphic classification^1^, ^3^, ^4^ showed 5 patients with columnar type (16.1%), 5 patients with cystic type (16.1%), and 21 patients with spindle type (67.7%) (Fig. 1a) (Table 3). Associated anomalies included APBDU in seven patients and PD in four patients. Concomitant pathologies included choledocholithiasis in three patients, cholelithiasis in six patients, sickle cell disease in one patient, and common bile duct cancer in one patient (Table 3). Of the 31, 15 (48%) Type I patients underwent an operation, including 4 who had choledochectomy and hepaticojejunostomy, 3 who had cholecystectomy with choledochoduodenostomy, 3 who had major sphincterotomies, and 5 who had a cholecystectomy alone. Thirteen patients were observed nonoperatively, and three patients were lost to follow up.

**Table 3:** Choledochal cyst (Type I)

Cases	31 (0.56%; 31/5522)	
Age	8–80	
Gender	Male 10, Female 21	
Form	Columnar type (a)	5
	Cystic type (b)	5
	Spindle type (c)	21
Associated conditions	APDU	7†
	Choledocholithiasis	3
	Cholelithiasis	6
	Pancreas divisum	4
	Sickle cell disease	1
	Common Bile Duct Cancer	1

† Duplicate.

**Figure 2:**
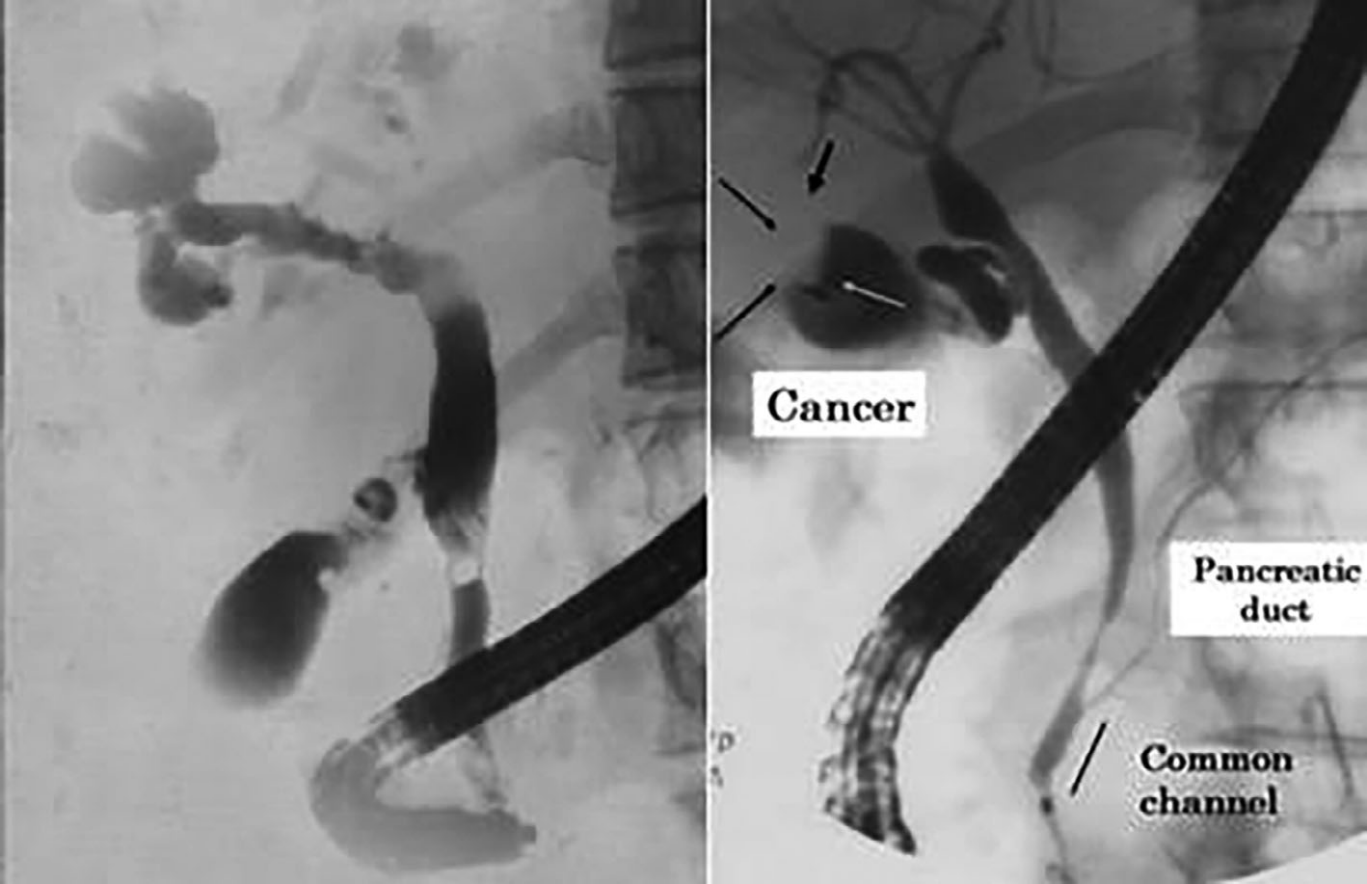
(a) Caroli's disease (type V): Intrahepatic cysts. (b) Anomalous pancreaticobiliary ductal union (APBDU). A P‐B type where the pancreatic duct joined the common bile duct. This patient had gallbladder cancer (arrow) without a dilated bile duct.

There were nine patients with Todani's Type III choledochocele (Table 1) (Fig. 1b). Six of the nine patients were treated with endoscopic sphincterotomy (Fig. 1b), two patients were treated by transduodenal cyst excision and surgical sphincteroplasty, and one patient was managed nonoperatively. The four patients with Todani's Type V Caroli's disease (Fig. 2a) were managed nonoperatively.^5^

### *Anomalous pancreaticobiliary ductal union*

APBDU is a congenital malformation of the confluence of the pancreatic and bile duct with the absence of a septum between the ducts.^4^ With APBDU, the common bile duct and pancreatic duct are joined outside of the duodenal wall proximal to the sphincter of Oddi (Fig. 1a). The 20 patients (0.36% of all ERCP patients) with APBDU ranged in age from 8 to 80 years, and there were 8 men and 12 women (Table 4). Associated anomalies included CCs in seven patients, which included five Type Ic and two Type Ia, according to Todani's classification.^1^ Two patients had PD. Other concomitant pathologies included three patients with cholelithiasis, two patients with sickle cell disease, three patients with pancreatitis, one patient with choledocholithiasis, and one patient with gallbladder cancer (Fig. 2b). APBDU was classified into three types: a B‐P type in which the insertion of the bile duct is into the pancreatic duct; a P‐B type where the pancreatic duct appears to join the common bile duct (Fig. 2b); and a Y type, in which there is a long common channel.^3^, ^6^ Of the 20 patients with APBDU, 9 had a P‐B‐type union (45%), 5 patients had B‐P union (25%), and 6 patients had a Y‐type union (30%). Of these 20 patients, 8 underwent an operation, which included 3 cholecystectomies, 1 cholecystectomy with lymphadenectomy for gallbladder cancer (Fig. 2b), 2 endoscopic sphincterotomies, and 2 choledochectomies with hepaticojejunostomy for Type I CC. The remaining 12 patients were managed nonoperatively.

**Table 4:** Anomalous pancreaticobiliary ductal union

Cases	20 (0.36%)
Age	8–80 years
Gender	Male 8 Female 12
Form	Nondilated: 13 cases
	Dilated: 7 cases

Associated conditions	Cases
Choledochal cyst (Type I)	7†
Cholelithiasis	3
Pancreas divisum	2†
Sickle cell disease	2
Pancreatitis	3
Choledocholithiasis	1
Gallbladder cancer	1

† Duplicate.

### *Other pancreaticobiliary anomalies*

**Figure 3:**
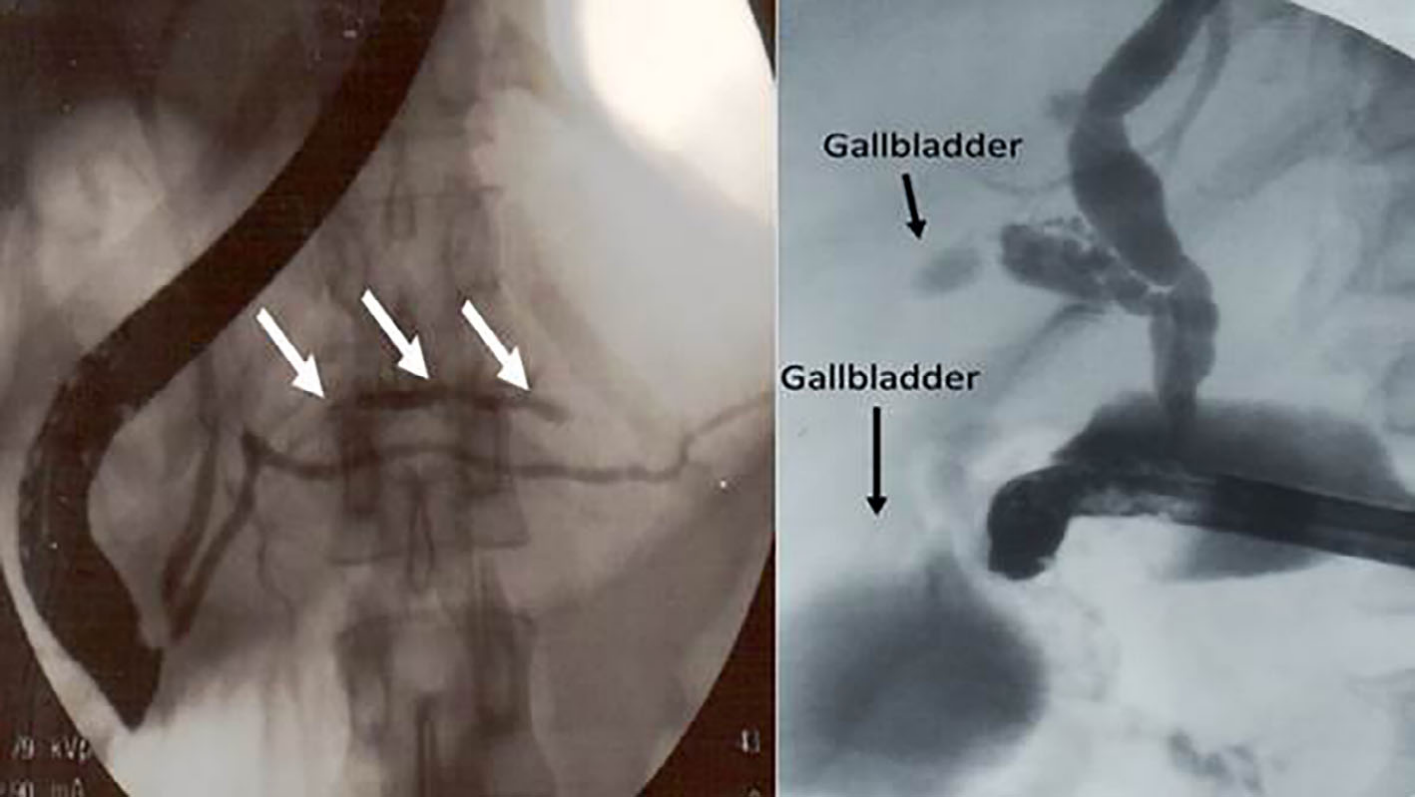
(a) (Pancreas Bifidum)—Double main pancreatic duct (arrow). (b) Double gallbladder (arrows).

Six patients (0.36% of ERCP patients) had other anomalies (Table 1). These included two patients with pancreas bifidum (Fig. 3a) and one patient each with a duplication of the gallbladder (Fig. 3b), a cystic duct diverticulum, an annular pancreas, and an abnormal cystic duct origin. Of the two patients with pancreas bifidum, one was treated nonoperatively, and 1 required a cholecystectomy. The single patient with a duplication of the gallbladder was treated by removal of both gallbladders. The patient who had cystic duct diverticulum was treated by cholecystectomy with diverticulectomy. The patient with an annular pancreas was treated by gastrojejunostomy, and the patient who had an abnormal cystic duct origin was treated by cholecystectomy.

## Discussion

Pancreaticobiliary anomalies commonly arise from abnormal development during gestation. The liver, biliary system, and pancreas are formed in the third to seventh week of gestation. During the fourth week of gestation, two endodermal buds arise from the duodenum as the hepatic diverticulum and dorsal pancreatic bud. The hepatic diverticulum evolves into the liver, intra‐ and extrahepatic biliary system, gallbladder, and ventral pancreas. During the fourth week of gestation, a ventral bud from the hepatic diverticulum fuses with the dorsal pancreatic bud, thus forming the head and uncinate process of the pancreas. A larger dorsal pancreatic bud cranial to the hepatic diverticulum forms the body and tail.^6^, ^7^

The biliary ducts are formed by endodermal cell proliferation and migration into mesodermal hepatic cells. These form a web‐like configuration composed of multiple connections with the gallbladder, cystic duct, and extrahepatic bile ducts.^3^ These extra‐anatomic connections are normally obliterated as the mature bile duct configuration is recannulated.^4^ The pancreatic ducts are formed by the separate dorsal and ventral buds. The dorsal pancreas forms the duct of Santorini with its minor papilla, and the ventral pancreas forms the duct of Wirsung and the major papilla. Failure of one or more of these events to occur leads to various anatomic anomalies.^6^, ^7^

PD results from the failure of the ducts of the dorsal and ventral pancreatic buds to fuse properly, causing an aberrant communication between the two ductal systems. This failure can be subdivided into complete and incomplete. Complete divisum occurs when there is no communication between the ductal systems, and an incomplete divisum occurs when the communication is through only a small communicating branch. The frequency at which these variants occur varies, depending on the patient group, but the clinical implications appear to be similar for each variant because the dorsal duct drains the majority of the gland in these instances.^8^ PD has been named as a cause of pancreatitis due to a narrowing of the opening of the minor papilla. This is thought to cause an inadequate outflow of pancreatic fluids. PD has been reported to be found in 5.7% of patients undergoing ERCP.^9^ The infrequency of this anatomic anomaly plus the many common causes of pancreatitis make it difficult to prove a causative relationship.^8^, ^9^, ^10^, ^11^, ^12^ Our data showed that 59 of the 179 patients with PD had pancreatitis. Many of these, however, reported an excessive use of alcohol. Furthermore, 26 of these patients had cholelithiasis or choledocholithiasis. Therefore, it is not possible to define a direct causative relationship.^12^ A recent study^13^ has shown an association between some genetic abnormalities and PD. Bertin has recently shown that mutations in the cystic fibrosis transmembrane conductance regulator gene (CFTR), the serine protease inhibitor Kazal type 1 gene (SPINK1), and the cationic trypsinogen gene (PRSS1) are associated with both chronic pancreatitis and acute recurrent pancreatitis. These genetic alterations likely resulted in increased viscosity of the pancreatic fluid, which may clog smaller ductal systems and predispose these individuals to pancreatitis. Although not completely understood, a multifactorial model is likely to evolve to explain how pancreatitis occurs in individuals with PD that includes both anatomic changes and a variety of individual predisposing genetic factors.^7^, ^13^

The treatment for PD is controversial. Patients with well‐defined idiopathic pancreatitis are more likely to benefit from endoscopic minor papillotomy or surgical sphincteroplasty and/or stenting.^5^, ^11^, ^14^, ^15^ Surgical transduodenal minor papillary sphincteroplasty followed by endoscopic stenting may also be helpful.^16^ Some studies have reported improved symptomatic relief in patients that underwent surgical *versus* endoscopic treatment for PD due to a larger orifice that can be achieved surgically.^17^ In our study, eight patients were candidates for sphincterotomy (Table 2) for recurrent acute pancreatitis. Finally, most cancer patients with PD had malignancies involving the common duct, ampulla, or pancreatic head; operations in these patients were directed toward cancer treatment (Table 2).

Congenital choledochal cysts may arise in association with pancreatic anomalies. Most CCs are Todani's classification Type I (Fig. 1a),^1^, ^3^, ^4^, ^18^ also known as congenital biliary dilatation. This is composed of a fusiform saccular extrahepatic biliary dilation involving the common bile duct and common hepatic duct. These are usually resected and followed by Roux‐en‐Y hepaticojejunostomy.^18^, ^19^, ^20^ The incidence of a Type I CC worldwide is thought to be 1:2 000 000,^5^ four times more common in Asian populations, and there is a 3:1 female‐to‐male ratio.^3^, ^5^, ^18^, ^19^, ^20^ This is primarily a disease of pediatric patients who usually present with the triad of abdominal pain, jaundice, and abdominal mass.^21^ Complications of Type I CC include cholelithiasis, bile duct cancer, pancreatitis, biliary cirrhosis, cyst rupture, and liver abscess.^18^, ^19^, ^20^, ^21^, ^22^ The etiology is thought to be failure of migration of pancreaticobiliary junction to the duodenal luminal wall, resulting in a long anomalous pancreaticobiliary channel or persistent distal obstruction causing dilation and cyst formation (Fig. 1a).^22^ CCs may also have a genetic predisposition.^23^, ^24^, ^25^ Amongst our data, predominant Todani's classification was Type I, and these were found to be associated most commonly with APBDU, which is consistent with prior reports.

In addition, we did find nine patients with Type III cysts and four patients with Type V cyst. Type III cysts commonly present with abdominal pain as the predominant symptom, followed by nausea, vomiting, fever, and jaundice.^26^ These are often initially diagnosed via ultrasound or computed tomography (CT) scan and further studied and treated via ERCP. Endoscopic therapies include sphincterotomy (Fig. 1b), cyst marsupialization, and stent placement.^4^, ^26^, ^27^ Surgical management is uncommon, largely due to the low risk of malignant transformation with Type III cysts.^4^, ^26^ Type V cysts (Caroli's disease) (Fig. 2a) are usually treated via a variety of endoscopic and surgical treatments. Surgical options include segmental resection if only a portion of the liver is involved or possible transplantation should there be diffuse involvement. Among the patients who are amenable to partial hepatic resection, many will continue to require endoscopic interventions for the remaining portion of the liver. In addition, the risk of malignant transformation is higher and increases with age in patients with Type V cysts.^3^

With APBDU, the common bile duct and pancreatic duct are joined outside of the duodenal wall proximal to the sphincter of Oddi.^3^, ^6^, ^28^ The sphincter of Oddi normally encompasses the common channel and both the common bile duct and pancreatic duct. Normal function of the sphincter prevents pancreatic secretions from refluxing into the biliary tree. In addition, there are a few variations of APBDU, depending on whether the pancreatic duct appears to be entering the common bile duct (P‐B) (Fig. 2b), the common bile duct appears to be entering the pancreatic duct (B‐P), or if there is a long common channel measuring greater than 15 mm in length (Y type).^6^ In a large series, the B‐P and P‐B types have each been reported to be the most common type of APBDU.^6^, ^28^

There is a proven relationship between APBDU and biliary tract cancers. The reflux of pancreatic secretions and biliary stasis causes chronic inflammation that subsequently induces hyperplasia and metaplasia, thus contributing to an increased risk of bile duct and gallbladder carcinoma.^29^, ^30^, ^31^, ^32^ The bile from patients with APBDU promotes the proliferation of cholangiocarcinoma cells through an induction of the COX‐2 inflammatory pathway.^33^ The type of cancer (bile duct *vs* gallbladder), the incidence of each, and age of onset appear to vary depending on whether biliary dilatation is present.^28^, ^34^ Gallbladder cancer has the highest occurrence if bile duct dilatation is not present (Fig. 2b) and is thought to be due to greater pressures causing increased reflux to the level of the gallbladder.^28^, ^34^

Current recommendations for management of patients with APBDU involve surgical treatment due to increased risks of malignancy. In patients with a common bile duct dilatation, it is recommended that they undergo prophylactic cholecystectomy with extrahepatic bile duct resection. However, in patients without common bile duct dilatation, it is being debated whether prophylactic cholecystectomy is enough as gallbladder cancer far outweighs incidence of bile duct cancer in these patients.^28^, ^34^ Moreover, ERCP can be used as a bridge to definitive surgical management in patients who present with acute symptoms. The cause of acute pain and pancreatitis in this patient population is thought to be due to protein plugs in the common channel. ERCP can not only relieve these obstructions and provide better outflow by means of a sphincterotomy but can also image the pancreaticobiliary system to provide the needed information for surgical planning.^35^, ^36^ A recent article states that extrahepatic bile duct resection is the standard surgery for congenital biliary dilatation.^37^ However, complete excision of the intrapancreatic bile duct and removal of stenotic hepatic ducts are necessary to prevent serious complications, including recurrent cancer after surgery. In adults with APBDU and no dilated bile duct, excision of the common bile duct is regarded as unnecessary because bile duct carcinomas rarely develop. Surgical management of pancreaticobiliary maljunction and congenital biliary dilatation might undergo major changes in the future.^37^

Sphincter of Oddi stenosis^38^ can be difficult to differentiate from the congenital Type Ic CCs (spindle type with or without APBDU). Endoscopic sphincterotomy is recommended for sphincter of Oddi stenosis.^38^ Endoscopic biliary sphincterotomy appears to be a logical step in the management of most symptomatic patients with a Type Ic CC.^2^, ^35^, ^36^, ^38^

With regard to the other pancreaticobiliary anomalies, most variations in the anatomy of the biliary tree appear to result from alterations in the budding from the foregut of the embryonic biliary tract. Minor anomalies usually cause no clinical problems but may be of great relevance to the biliary surgeon. An accessory bile duct from the right hepatic duct to the cystic duct or gallbladder (duct of Luschka) may result in biliary leak postcholecystectomy. Pancreas bifidum is a very rare congenital anomaly of the main pancreatic duct. On magnetic resonance cholangiopancreatography (MRCP) or ERCP, it manifests as a duplication of the major duct in the body of the pancreas (Fig. 3a) and is usually benign and incidentally detected.^39^

In summary, this study highlights incidence, symptoms, and treatment of pancreatic and biliary anomalies observed by a single endoscopist over a 43‐year time period at an inner‐city medical center. Abdominal pain is the most common presenting symptom, which is vague and often results in delayed diagnosis and treatment. Therefore, the treating physician must be aware of these anomalies to facilitate accurate and timely diagnosis and treatment.
